# What it Takes to Successfully Implement Technology for Aging in Place: Focus Groups With Stakeholders

**DOI:** 10.2196/jmir.5253

**Published:** 2016-05-03

**Authors:** Sebastiaan Theodorus Michaël Peek, Eveline JM Wouters, Katrien G Luijkx, Hubertus JM Vrijhoef

**Affiliations:** ^1^ Institute of Allied Health Professions Chair of Health Innovations and Technology Fontys University of Applied Sciences Eindhoven Netherlands; ^2^ School of Social and Behavioral Sciences Department of Tranzo Tilburg University Tilburg Netherlands; ^3^ Saw Swee Hock School of Public Health National University of Singapore Singapore Singapore; ^4^ National University Health System Singapore Singapore

**Keywords:** aged, independent living, technology, eHealth, health services for the elderly, project and people management, implementation management, qualitative research, focus groups

## Abstract

**Background:**

There is a growing interest in empowering older adults to age in place by deploying various types of technology (ie, eHealth, ambient assisted living technology, smart home technology, and gerontechnology). However, initiatives aimed at implementing these technologies are complicated by the fact that multiple stakeholder groups are involved. Goals and motives of stakeholders may not always be transparent or aligned, yet research on convergent and divergent positions of stakeholders is scarce.

**Objective:**

To provide insight into the positions of stakeholder groups involved in the implementation of technology for aging in place by answering the following questions: What kind of technology do stakeholders see as relevant? What do stakeholders aim to achieve by implementing technology? What is needed to achieve successful implementations?

**Methods:**

Mono-disciplinary focus groups were conducted with participants (n=29) representing five groups of stakeholders: older adults (6/29, 21%), care professionals (7/29, 24%), managers within home care or social work organizations (5/29, 17%), technology designers and suppliers (6/29, 21%), and policy makers (5/29, 17%). Transcripts were analyzed using thematic analysis.

**Results:**

Stakeholders considered 26 different types of technologies to be relevant for enabling independent living. Only 6 out of 26 (23%) types of technology were mentioned by all stakeholder groups. Care professionals mentioned fewer different types of technology than other groups. All stakeholder groups felt that the implementation of technology for aging in place can be considered a success when (1) older adults’ needs and wishes are prioritized during development and deployment of the technology, (2) the technology is accepted by older adults, (3) the technology provides benefits to older adults, and (4) favorable prerequisites for the use of technology by older adults exist. While stakeholders seemed to have identical aims, several underlying differences emerged, for example, with regard to who should pay for the technology. Additionally, each stakeholder group mentioned specific steps that need to be taken to achieve successful implementation. Collectively, stakeholders felt that they need to take the leap (ie, change attitudes, change policies, and collaborate with other organizations); bridge the gap (ie, match technology with individuals and stimulate interdisciplinary education); facilitate technology for the masses (ie, work on products and research that support large-scale rollouts and train target groups on how to use technology); and take time to reflect (ie, evaluate use and outcomes).

**Conclusions:**

Stakeholders largely agree on the direction in which they should be heading; however, they have different perspectives with regard to the technologies that can be employed and the work that is needed to implement them. Central to these issues seems to be the tailoring of technology or technologies to the specific needs of each community-dwelling older adult and the work that is needed by stakeholders to support this type of service delivery on a large scale.

## Introduction

A key challenge for most, if not all, countries is how to accommodate and care for an aging population [1]. As a response, many countries have shifted their priorities and resources toward deinstitutionalization in order to create communities that facilitate seniors to remain living in their homes for as long as possible [2]. Policies and programs that represent this paradigm shift frequently emphasize the deployment of technology as a means of supporting aging in place. Examples of technologies mentioned are sensor-based networks for activity monitoring, emergency help systems, and online tools to support older adults’ self-management of chronic conditions [3,4]. These technologies are often information and communications technology (ICT) based; they are referred to as eHealth, ambient assisted living technology, smart home technology, and/or gerontechnology. Unfortunately, the implementation of these technologies is frequently unsuccessful in daily practice [5-7].

Several factors hinder the implementation of the aforementioned technologies, including low adoption levels among potential users [3,4,7,8], difficulties in building sustainable business cases [9,10], a lack of interoperability between systems of different vendors [6,9,11], and scarcity of robust scientific evidence on cost and outcomes [12-14]. All the aforementioned factors are complicated by the fact that multiple stakeholders are involved [9,15]. Typical stakeholders include older adults, care professionals, managers within home care or social work organizations, technology designers and suppliers, and policy makers. The goals and motives of these groups of stakeholders may not always be transparent or aligned [16,17]. However, empirical studies providing insight into the convergent and divergent perspectives of stakeholders involved in implementing technology that could support aging in place are few and far between. Furthermore, the few existing studies limit their focus on perceived barriers to a successful implementation [18,19] rather than forming a more complete understanding of stakeholders’ positions. For example, several authors have noted that it is crucial to understand what the different stakeholders’ goals are in initiatives centered around supporting aging in place with technology [20-22]. Hence, this study seeks to provide insight into the positions of stakeholder groups involved in the implementation of technology for aging in place by asking the following three questions: What kind of technology do they see as relevant for aging in place? What do they aim to achieve by implementing technology? What is needed to achieve successful implementations? A better understanding of the positions of various stakeholder groups is expected to contribute to the successful implementation of technological interventions aimed at supporting aging in place [11,20,23,24].

## Methods

### Sampling

This study was conducted in the Netherlands. In 2012, our research group, in collaboration with 13 partners, initiated a project aimed at finding ways to successfully deploy technologies that could support aging in place, by conducting a longitudinal field study among community-dwelling older adults. As a part of the project, five mono-disciplinary focus groups were conducted simultaneously with participants representing five groups of stakeholders within the process of implementing technology for aging in place: older adults, care professionals, managers within home care or social work organizations, technology designers and suppliers, and policy makers. These focus group sessions took place in February 2012, and convenience sampling was used by the partners of the project to recruit participants. This means that participants in the focus groups were either working for one of the partners in the project or were professional relations of partners. At the time the focus group sessions were conducted, participants representing different stakeholder groups were not engaged in implementing technology for aging in place together. Mono-disciplinary focus groups were employed because this data collection method was expected to efficiently enable productive discussions and the elicitation of a multiplicity of views by each stakeholder group [25]. Furthermore, we wanted to provide a safe environment for participants [25].

### Procedure

Focus group sessions took place simultaneously in the Fontys Institute of Allied Health Professions, which is located in Eindhoven, the Netherlands. Sessions lasted 90 minutes and each session was supervised by a moderator and an assistant. Moderators had a professional background that was related to the background of the participants in their session. At the beginning of the sessions, a scenario was read out loud by the moderators. The scenario described how population aging increases the need for creative solutions to be able to continue to provide good quality care for older adults. Furthermore, the scenario explained that more and more older adults are expected to age in place, and that technological solutions are expected to play an important role in this respect. In the group discussion that followed within each session, three open-ended questions were discussed by participants. First, participants were asked what kinds of technologies they considered as "technologies that could support aging in place". This question was asked to make transparent what stakeholders perceived as technology relevant to the context of aging in place. Second, participants were asked when they would consider the use of technology for aging in place a success. This was asked to determine what stakeholders are trying to achieve with regard to the implementation of technology for aging in place. Third, participants were asked what they need to be able to successfully implement the technology for aging in place, and what they can contribute in order to achieve successful implementations. This was done to let participants reflect on their role as stakeholders. After each question, participants were first requested to write down their answers on a form to enable them to collect their thoughts prior to engaging in the discussions. Informed consent was acquired from all participants and each session was recorded by audio and video to enable transcription. Transcripts were made anonymous and all data was only used in this study. Dutch law does not require medical or ethical reviews for focus group interviews with stakeholders other than patients. All moderators were trained according to guidelines described by Sim [25] and provided with a guide that was produced by the lead author. Each moderator was accompanied by an assistant who took notes and aided in facilitating an open dialogue between group members. Immediately after the sessions, the moderators and assistants gathered to evaluate the discussions. The moderator and assistant of the session that consisted of technology designers and suppliers stated that they had to intervene regularly because some participants were dominant in the discussion, and because participants needed to be reminded to reflect on their own role instead of focusing on the role of other stakeholders. Moderators and assistants of the other group sessions did not experience these issues, or to a far lesser extent.

### Analysis

Verbatim transcripts of the sessions were analyzed using thematic analysis [26]. First, inductive codes were attached to quotations relevant to the research questions. In this process, each transcript was initially coded independently by two researchers, who subsequently had to come to an agreement and produce a single coded version of each transcript. Afterward, overarching categories of codes (ie, themes and subthemes) were formed. Additionally, the technologies that the participants deemed relevant for aging in place were classified in application domains that are part of the gerontechnology taxonomy as proposed by van Bronswijk, Bouma, and Fozard [27]. This taxonomy was selected because it is targeted toward technologies that are relevant to older adults and because it allows for the inclusion of a wide range of technologies, which is in line with the participants’ responses. As a member check, a separate meeting was organized in which preliminary findings were presented. In this way, participants were provided with the opportunity to learn more about the positions of the various stakeholder groups involved in the project. Two-thirds of the participants attended the meeting and they accepted the presented findings as accurate and complete.

## Results

### Participants

A total of 29 participants were involved in the study and each stakeholder group was represented by 5-7 participants (see Table 1). Participants were 32-76 years old, and the average age was highest in the focus group with older adults. The managers in the study were all women. Care professionals were predominantly women, while technologists were predominantly men.

**Table 1:** Stakeholders and participants involved in mono-disciplinary focus groups (n=29).

Stakeholder	Description of participants	Participant characteristics	n (%)
Older adults (O)	Community-dwelling older adults (active in community voluntary work)	3 men and 3 women, aged 62-76 years	6 (21)
Care professionals (C)	Care professionals who provide home care themselves, or coordinate the provision of home care	1 man and 6 women, aged 32-55 years	7 (24)
Managers (M)	Managers within home care or social work organizations	5 women, aged 37-61 years	5 (17)
Technologists (T)	Professionals who work for companies that produce and supply technology, or for educational institutions with a focus on technology	5 men and 1 woman, aged 36-66 years	6 (21)
Policy makers or advisors to policy makers (P)	Public officers, and advisors and researchers involved in health policy	3 men and 2 women, aged 32-61 years	5 (17)

**Table 2:** Technology believed to play a role in supporting aging in place according to stakeholder groups, categorized in application domains as proposed in the gerontechnology taxonomy.

Application domains	Technologies	O^a^	C^b^	M^c^	T^d^	P^e^
**Health and self-esteem**						
	Health monitoring	X^f^	X	X	X	X
	Personal alarms	X	X	X	-^g^	X
	Physical activity stimulation	X	-	-	X	X
	Fall detection	-	X	X	-	X
	Medication reminders	-	-	X	X	X
	Wandering detection	-	-	X	X	-
	Online questionnaires	X	-	-	-	X
	Lifestyle monitoring	-	-	-	X	-
**Housing and daily living**						
	Assistive technology	X	X	X	X	X
	Home automation	X	X	X	X	X
	Household appliances	X	X	X	X	X
	ADL^h^robots	X	-	X	X	X
	Electronic agendas	X	-	-	-	X
	Home adaptations	-	X	-	X	-
	Lift assist devices	-	-	-	X	-
**Communication and governance**						
	Computers	X	X	X	X	X
	Video telephony	X	X	X	X	X
	Caregiver e-collaboration	X	-	X	X	X
	Electronic health records	X	-	X	-	-
	Social media	-	-	X	-	X
	Telephones	X	-	X	-	-
**Work and leisure**						
	Television and radio	X	-	X	-	X
	E-readers	X	-	-	X	-
	Games	-	-	-	-	X
**Mobility and transport**						
	Transportation devices	X	-	X	X	-
	GPS^i^navigation	-	-	-	X	-

^a^O: older adults.

^b^C: care professionals.

^c^M: managers.

^d^T: technologists.

^e^P: policy advisors and policy makers.

^f^X: mentioned by stakeholder group.

^g^-: not mentioned by stakeholder group.

^h^ADL: activities of daily living.

^i^GPS: global positioning system.

**Table 3:** Stakeholders’ perspectives on what constitutes successful implementation of technology for aging in place: major themes, subthemes, and typical quotations.

Major themes	Subthemes	Illustrative quotations	O^a^	C^b^	M^c^	T^d^	P^e^
**User-centeredness: Older adults’ needs and wishes are given priority during development and deployment of the technology, meaning...**							
	...the technology is in accordance with each older adult’s specific needs.	“What’s needed is a solution for what the individual thinks is a problem, not what we consider a problem” (P #4)	X^f^	X	X	X	X
	...older adults are in control.	“So that it’s not the technology that controls my life, but rather it’s me controlling the technology” (O #6)	X	X	X	-^g^	-
	...older adults’ privacy is treated with respect.	“Seniors shouldn’t get the feeling they’re being followed or watched” (C #6)	X	X	-	X	-
**Acceptance: The technology is accepted by older adults, meaning...**							
	...older adults enjoy using the technology.	“A positive experience, causing people to use it again” (M #1)	X	X	X	X	X
	...the technology is used on a regular basis.	“When technology is actually being used” (P #3)	-	X	X	X	X
	...older adults are proud to use the technology (instead of ashamed).	“It shouldn’t be stigmatizing” (O #6); “I feel we should aim to create a hype” (M #4)	X	-	X	X	X
**Benefits: Use of the technology provides benefits to older adults, meaning...**							
	...the technology improves the quality of life of older adults.	“When the client or individual experiences that his or her quality of life remains the same or increases markedly” (M #5)	X	X	X	-	X
	...the technology supports independent living.	“If no one needs to go to a nursing home” (T #2)	-	X	X	X	-
	...the technology provides reassurance.	“Causing people to find an answer to a slowly rising fear of being unstable, frail" (T #5)	X	X	-	X	-
**Prerequisites: Favorable prerequisites for ownership and use of technology by older adults exist, meaning...**							
	...the technology is easy to use.	“The technology must be extremely user friendly” (M #2)	X	X	X	X	X
	...the technology is affordable.	“Affordability continues to be a problem” (T #6)	X	X	X	X	X
	...the technology is reliable.	“It must work, it must be reliable” (O #3)	X	X	-	X	-
	...technical support is available.	“The supplier or care organization must provide good service” (O #3)	X	X	-	-	X

^a^O: older adults.

^b^C: care professionals.

^c^M: managers.

^d^T: technologists.

^e^P: policy advisors and policy makers.

^f^X: mentioned by stakeholder group.

^g^-: not mentioned by stakeholder group.

### Types of Technology That Could Support Aging in Place

Stakeholders had a broad view with regard to technology that could support aging in place, which in their eyes included hardware, software, or combinations of both. In addition, technologies that are not based on ICT were mentioned (eg, consumer appliances and home adaptations). The technologies that were mentioned can be classified in application domains that are part of the gerontechnology taxonomy [27]: health and self-esteem, housing and daily living, mobility and transport, communication and governance, and work and leisure (see Table 2).

In total, 26 different technologies were mentioned by stakeholders across the five domains of the gerontechnology taxonomy. These technologies for the most part fall under the domains of health and self-esteem (8/26, 31%), housing and daily living (7/26, 27%), and communication and governance (6/26, 23%). Out of the 26 technologies mentioned, 5 (19%) fall under the domains of work and leisure or mobility and transport. Care professionals in total mentioned 9 out of 26 (35%) different types of technology, while the other stakeholder groups each mentioned 17 out of 26 (65%) different types. Out of the 26 technologies, 6 (23%) were mentioned by all stakeholder groups—health monitoring, assistive technology, home automation, household appliances, computers, and video telephony—while 3 (12%) technologies—lifestyle monitoring, lift assist devices, and global positioning system (GPS) navigation—were mentioned by one stakeholder group, the technologists. All other technologies were mentioned by two, three, or four stakeholder groups.

### Opinions on What Constitutes a Successful Implementation of Technology

All stakeholder groups considered the implementation of technology for aging in place a success when older adults’ needs and wishes are prioritized during development and deployment of technology, the technology is accepted by older adults, the technology provides benefits to older adults, and favorable prerequisites for the use of technology by older adults exist (see Table 3). According to the participants, the aforementioned four major themes—user-centeredness, acceptance, benefits, and prerequisites—are interrelated. All stakeholder groups stressed the importance of taking the perspective of older adults into account, and there was a shared belief that such a user-centered approach would have a positive effect on the acceptance of technology, on the benefits technology can provide, and on the existence of favorable conditions for technology use. Moreover, there was a common belief that technology can only provide benefits to older adults when it is accepted by them, and that acceptance of technology is dependent on certain prerequisites that need to be in place. A typical example of this notion is the following quotation: “Low ease of use leads to nonuse and a lack of added value” (Policy maker/policy advisor #5).

Looking at the first major theme, *user-centeredness*, and its underlying subthemes, all stakeholder groups found it important that technology is in line with the needs of each specific older individual. For example, older adults and policy makers mentioned that technology should not stand in the way of human contact. User-centeredness was also reflected in the fact that stakeholders mentioned that older adults need to be in control over technology instead of the other way around, and that the privacy of older adults needs to be treated with respect. However, policy advisors, care professionals, and older adults also stated that individual differences can make it difficult or expensive for technology to meet older adults’ needs in every situation: “It’s very hard to achieve this technically...how many diseases are there, and how many different impairments? Think about it” (Older adult #4).

The second major theme, *acceptance*, implicates that older adults enjoy using the technology, and that they use it on a regular basis. It also means that older adults are proud to use technology. The latter point reveals a difference of tone between stakeholder groups. Older adults stressed the importance of not feeling ashamed or stigmatized, while managers, technologists, and policy advisors talked in terms of taking pride in the technology: “It’s okay to have it in your home and show it to visitors: ‘Look what I have!’...It’s not all bad when you grow older, of course you want to show off the nice things that you have” (Technologist #3).

With regard to the third major theme, *benefits*, and its underlying subthemes, stakeholders felt that technology needs to improve older adults’ quality of life, support their ability to live independently, and provide reassurance (ie, enhance safety). However, care professionals, managers, and policy advisors stressed that other stakeholder groups are also involved in using technology for aging in place: “People often look at older adults as being the end user. However, informal and professional caregivers are also end users” (Policy advisor/policy maker #2). According to managers, this implies that professional caregivers need to see the benefits of employing technology as well. Older adults felt that technology should provide benefits, but also that technology should not make life too easy: “I think that technology should not make people lazy. For instance, mobility scooters—with all due respect for people who need them—are being used too easily, causing people to walk less” (Older adult #6).

The fourth major theme, *prerequisites*, entails the existence of conditions favorable to technology use and ownership. More specifically, stakeholders mentioned that technology should be easy to use, affordable, and reliable. Additionally, technical support should be available, preferably in person: “I think that there should be a physical location where one can ask something...personal support” (Policy advisor/policy maker #5). Care professionals and technologists, especially, expressed concerns with regard to affordability. Care professionals mentioned that technology in care settings can be expensive, and they worry about who would pay for the technology. Technologists mentioned that they foresee a trend where older adults themselves are the ones who pay for technology. In this scenario, technologists see older adults’ willingness to pay for technology as critical, and they feel that the technology they wish to sell needs to be more affordable than competing alternatives. In contrast, older adults only fleetingly mentioned the fact that technology needs to be affordable. As for managers, they looked at affordability from a cost-benefit perspective: “When the financial benefits exceed the investments” (Manager #1).

### What is Needed to Successfully Implement Technology for Aging in Place

Looking at their own roles, stakeholders mentioned several things that they need or can contribute to enable successful implementations of technology for aging in place. These can be organized into four major themes and eight underlying subthemes (see Table 4).

**Table 4:** Stakeholders’ views on what is needed to successfully implement technology for aging in place: major themes and subthemes.

Major themes	Subthemes	O^a^	C^b^	M^c^	T^d^	P^e^
**Take the leap**						
	Change in attitude(s)	X^f^	X	X	X	-^g^
	Change in policies	-	X	X	X	X
	Collaborate with other organizations	-	-	X	-	X
**Bridge the gap**						
	Match technology with individuals	-	X	X	-	X
	Stimulate interdisciplinary education	-	-	-	-	X
**Facilitate technology for the masses**						
	Work on products and research that support large-scale rollouts	-	-	X	X	-
	Train target groups on how to use technology	X	X	-	-	-
**Take time to reflect**	Evaluate use and outcomes	-	X	-	-	X

^a^O: older adults.

^b^C: care professionals.

^c^M: managers.

^d^T: technologists.

^e^P: policy advisors and policy makers.

^f^X: mentioned by stakeholder group.

^g^-: not mentioned by stakeholder group.

The first theme, *take the leap*, is concerned with what is needed in terms of commitment by stakeholders. Most stakeholder groups emphasized that a change in attitude is needed on their part to achieve successful implementations. For example, older adults mentioned that they could be more assertive. By this, it was meant that older adults can improve in “Saying what you think, desire, and feel” (Older adult #5), and that older adults are prepared to ask for help. Older adults stated that this is particularly important when talking to technologists. Additionally, older adults mentioned that they sometimes need to be stimulated to use technology, or as one older adult phrased it, “Pushed gently” (Older adult #6). Reflecting on their own role, care professionals stated that they need to adjust, and accept that things are changing: “From a caring perspective, I want to help people in person...however, some things are no longer feasible. I feel that a new mindset is needed” (Care professional #7) and “It’s the client who has technology in his home, and we need to become accustomed to it” (Care professional #4). Managers felt that they need to promote the use of technology more. They mentioned that they could initiate pilot projects, which are seen as a way to have care professionals gain experience in using technology. Technologists mentioned that technology companies need to be prepared to take financial risks. More specifically, companies need to have the confidence to produce and roll out technologies on a large scale. For this, a long-term strategy and perseverance are required: “There can be up to 20 years between designing the thing, and starting to make a profit. We have to get used to that; that’s the long-term vision we have to have” (Technologist #3).

Additionally, most stakeholder groups proposed that policies need to be changed. Care professionals ask that the organizations that they work for formulate a privacy policy for situations in which technology is employed. Managers stated that they would like more flexibility with regard to the relevant laws and regulations. They also mentioned that they need to incorporate technology in their organizational strategy: “It all starts at the top: what are the priorities for the organization in the years to come? When technology isn‘t in there...” (Manager #5). Reflecting on their own roles, policy advisors and policy makers mentioned that a large proportion of technology for older adults is being subsidized, and that the use of these technologies is frequently not sustainable: “When the funding stops...the technology is no longer used” (Policy advisor/policy maker #2). They argue that they need to find ways to counter this unwanted effect of current policies. Some technologists noted that subsidizing technology may obscure the actual needs of potential clients: “When people receive something for free, I can’t make out whether they actually want it” (Technologist #1).

Furthermore, the need for more organizational collaboration was mentioned by managers and policy advisors. Managers within home care or social work organizations felt a need to collaborate with others outside of their own organization in order to enable successful implementations of technology for aging in place: “I can’t do it alone. I need the municipality, and collaboration with the housing association and welfare organizations. You have to combine forces” (Manager #4). In this respect, insurance companies, patients’ associations, and informal caregivers were also mentioned. Policy advisors and policy makers emphasized the importance of international and interdisciplinary collaboration.

The second theme, *bridge the gap*, entails the work that is needed to connect available technological solutions to the needs of each specific older adult. Care professionals, managers, and policy makers stated that help is needed to be able to match technology with individuals. Care professionals mentioned that they would benefit from a "decision tool." Such a tool should allow care professionals to find and select the appropriate technology or combination of technologies for each specific client. Ideally, the technologies and aids that are deployed should also be registered in electronic health records. The managers in the study, who worked for different organizations than the care professionals, also mentioned that they would like to provide the care professionals with such a decision tool. Moreover, managers stated they would like to work together with a person (ie, consultant) who knows which technologies are on the market, and who can match these with the problems older adults face while trying to maintain their independence. Policy makers and policy advisors felt that interdisciplinary education is required to achieve this: “Because you need to know what an individual needs, you have to understand that person, and subsequently you have to know how to arrange technologies, services, and care” (Policy advisor/policy maker #3).

With regard to the third theme, *facilitate technology for the masses*, managers and technologists discussed the need to engage in large-scale rollouts of technology. Managers stated that there is a demand for technological solutions that can benefit a large proportion of older adults. In their eyes, large-scale rollouts can increase the willingness of commercial companies to invest, which is seen as a requirement for making technology for aging in place affordable. In their perception, more research is needed to provide scientific evidence that technology for aging in place is effective, and this is also expected to increase support by the government. To be able to conduct large-scale rollouts, technologists mentioned that companies need to do more research in order to gain a more profound understanding of what drives or impedes technology use by older adults.

Additionally, comments were made with regard to empowering target groups to be able to take advantage of technology. Older adults stated that they need to attend courses to learn how to use technology when they are still healthy enough to attend them. Care professionals also mentioned that they need training to be able to work with the technology. In their eyes, this applies to inexperienced as well as experienced care professionals: “You have to let yourself get educated, particularly those of us who have been working for a long time” (Care professional #2).

The last theme, *take time to reflect*, entails the evaluation of use and outcomes. Care professionals mentioned that they see it as their responsibility to regularly evaluate whether the use of technology is appropriate and not too excessive: “You shouldn’t use technology for everything” (Care professional #5). Additionally, policy makers stated that they feel a need to measure whether the use of technology is successful in terms of the desired outcomes. They see it as their role to promote evidence-based solutions.

## Discussion

### Principal Findings

This study aimed to understand the positions of stakeholders who are involved in the implementation of technology for aging in place—older adults, care professionals, managers of care organizations, technologists, and policy makers. It was found that stakeholders considered a multitude of technologies to be relevant for enabling independent living. However, it is important to note that only a small number of technologies were mentioned by all stakeholder groups. Furthermore, care professionals mentioned considerably fewer different types of technology than other stakeholder groups, which is in line with previous research [8]. Additionally, studies have shown that older adults may not be aware of technologies that could be of benefit to them [28,29]. Therefore, when planning and initiating projects concerned with technological solutions for aging in place, it is advisable to take into account that stakeholders may have a limited understanding of the scope of available technologies, and that stakeholders may differ in their awareness of available technologies. Moreover, technologies that are not ICT based (eg, household appliances and home adaptations) are also relevant in the context of aging in place according to stakeholders. In this sense, their concept of technology is less exclusive than the commonly used definitions of ambient assisted living technology [30], smart home technology [6], and eHealth [31].

With regard to the aims of stakeholders, all stakeholder groups felt that the implementation of technology for aging in place can be considered a success when (1) older adults’ needs and wishes are prioritized during development and deployment of the technology, (2) the technology is accepted by older adults, (3) the technology provides benefits to older adults, and (4) favorable prerequisites for the use of technology by older adults exist. As such, all stakeholder groups were profoundly concerned with the position of older adults when it comes to implementing technologies for aging in place. This study aligns closely with work reported by Greenhalgh et al [32], in which the authors sought to define quality in the design, implementation, and use of telehealth and telecare solutions for older adults with assisted living needs. In that study, which involved older adults, technology suppliers, and service providers, it was concluded that every stakeholder needs to comprehend the (changing) needs and capabilities of older adults, as well as their social context [32]. Such an approach, centered around the older individual, also aligns with the trend toward patient empowerment and patient engagement [33-36]; technology may be used to empower seniors, but this requires their engagement during design and implementation.

While the stakeholders in this study generally appeared to have identical aims with regard to technology for aging in place, it is important to note that underlying differences existed between stakeholders. For example, all stakeholder groups agreed that technology should provide certain benefits to older adults, but older adults were the only group that stressed that technology should not provide too many benefits, since this could make people dependent on technology, which is in line with previous research [12,37,38]. Another example of the variance of opinion is affordability: stakeholders agreed that this is important, but they did not seem to be on the same page with regard to who should pay for the technology. Participants in this study were not involved in a joint effort to implement technology at the time that data for this study was gathered. Once stakeholders are further into the process of implementing technology together, the aforementioned differences in the interpretation of key aims, such as benefits and affordability, could lead to cases of *stakeholder dissonance*, which threatens a project’s viability if left undetected and unresolved [39].

Each stakeholder group mentioned specific steps that need to be taken to achieve successful implementations. Collectively, stakeholders felt they need to take the leap (ie, change attitudes, change policies, and collaborate with other organizations), bridge the gap (ie, match technology with individuals and stimulate interdisciplinary education), facilitate technology for the masses (ie, work on products and research that supports large-scale rollouts and train target groups on how to use technology), and take time to reflect (ie, evaluate use and outcomes). Some of the aforementioned steps or recommended actions have also been reported by similar stakeholder groups in other studies; for example, the need to focus on changing the attitudes of care receivers and care givers [40,41], the need to match technology with individual clients [28,40,42], and the need for training stakeholder groups [8,42,43]. Additionally, studies have pointed to recommended actions that were not mentioned by participants in this study. These include the need to consider how the introduction of technology affects the existing workflow in home care organizations [40-42] and the fact that care professionals require support while using technology [8,44,45].

The recommended actions brought forward by stakeholders in this study imply that structural changes need to be made on political/ strategic, organizational/ contractual, managerial/ scientific, and operational levels [46]. Such changes will not be easy to implement because of their fundamental character, and because they require changes in how different stakeholder groups operate and interface with one another [11,22,32]. Additionally, recent evaluations of the Delivering Assisted Living Lifestyles at Scale (dallas) program in England [11] and Scotland [22] indicate that while involving end users in the design of technologies could promote adoption, it is also very difficult to simultaneously codesign and deliver technologies at a large scale. The reason for this is that codesign is time and resource consuming [11,22]. This was also demonstrated by Linskell and Bouamrane [47], who described two possible routes for the delivery of technology that could support aging in place: a short and direct delivery route, which is prone to misinterpretation of user needs, and a longer codesign route which incorporates task analysis and more extensive specification of product requirements. Therefore, when it comes to matching technology with individuals, the challenge seems to lie in being able to determine when a short and direct delivery route is acceptable and when a longer codesign route is warranted.

The results of this study can be viewed in light of the Normalization Process Theory (NPT), as described by May and Finch [48-50]. NPT addresses “the factors needed for successful implementation and integration of interventions into routine work” [49] and consists of four main components: coherence (ie, meaning and sense making by stakeholders); cognitive participation (ie, commitment and engagement by stakeholders); collective action (ie, the work stakeholders do to make the intervention function in practice); and reflexive monitoring (ie, formal and informal appraisal of the benefits and costs of the intervention) [50]. Our findings seem to indicate that NPT can potentially provide a useful framework for studying implementations in the context of aging in place. First, the themes that emerged in this study with regard to what is needed to successfully implement technology for aging in place bear resemblance to NPT’s concepts of cognitive participation, collective action, and reflexive monitoring. For example, the *take the leap*   theme, which includes a change in attitudes, a change in policies, and collaboration with other organizations, resembles NPT’s cognitive participation component; the *bridge the gap*   and *facilitate technology for the masses*   themes are in line with NPT’s component of collective action. Second, NPT’s first component, coherence, includes a “shared understanding of the aims, objectives, and expected benefits” [51], and this study shows that focus group sessions can be employed to start to develop this type of shared understanding. However, it was not our goal to verify or test NPT in this study. Future studies are necessary to explore the value of NPT in the context of aging in place, particularly in situations where available technological solutions need to be matched to the specific needs of each client. Furthermore, focus group sessions in this study were mono-disciplinary and led to findings that pointed to several differences among stakeholder groups, indicating that it would be beneficial to follow up on these mono-disciplinary sessions by conducting heterogeneous sessions to further develop coherence.

### Limitations

Our study is limited by the fact that it may not have included all the relevant stakeholders. For example, research shows that family members and informal caregivers can play an important role in the (effective) use of technology by community-dwelling older adults [38,52]. Additionally, the grouping of stakeholders in this study is an oversimplification, as each stakeholder group can be broken down into more specific subgroups. Furthermore, process evaluations covering a longer period of time are needed to determine how dynamics between stakeholders influence the effective provisioning of personalized and appropriate technology that can help older adults to age in place. Lastly, it cannot be ruled out that our study was susceptible to selection bias since all participants were part of a project that aimed to improve the deployment of technology for aging in place by conducting research in the homes of older adults.

### Conclusions

In conclusion, this study adds to the limited body of work concerned with successfully implementing technology that aims to support aging in place. Stakeholders in this study largely agree on the direction in which they should be heading, yet they have different perspectives with regard to the technologies that can be employed and the work that needs to be done to implement these. Central to a successful implementation seems to be the tailoring of technology or technologies to the specific needs of each community-dwelling individual, and the work that is needed by stakeholders to support this type of service delivery on a large scale. Our findings indicate a tension between aiming to personalize technology implementations and aiming to deploy technology en masse. It is clear that, in order to successfully implement technology for aging in place, stakeholders need to engage in an ongoing mutual commitment focused on the goal of empowering older adults through the use of technology.

## Abbreviations

ADL: activities of daily living
C: care professionals
dallas: Delivering Assisted Living Lifestyles at Scale
GPS: global positioning system
ICT: information and communications technology
M: managers
NPT: Normalization Process Theory
O: older adults
OCW: Dutch Ministry of Education, Culture and Science
P: policy advisors and policy makers
RAAK: Regional Attention and Action for Knowledge Circulation
SIA: Stichting Innovatie Alliantie
T: technologists
